# Decreasing adrenergic or sympathetic hyperactivity after severe traumatic brain injury using propranolol and clonidine (DASH After TBI Study): study protocol for a randomized controlled trial

**DOI:** 10.1186/1745-6215-13-177

**Published:** 2012-09-26

**Authors:** Mayur B Patel, John W McKenna, JoAnn M Alvarez, Ayaka Sugiura, Judith M Jenkins, Oscar D Guillamondegui, Pratik P Pandharipande

**Affiliations:** 1 Veterans Affairs (VA) Tennessee Valley Healthcare System, Nashville VA Medical Center, 1310 24th Avenue South, Nashville, TN, 37212, USA; 2 Vanderbilt University Medical Center, Division of Trauma & Surgical Critical Care, Department of Surgery, 1211 21st Avenue South, 404 Medical Arts Building, Nashville, TN, 37212, USA; 3 Vanderbilt University School of Medicine, Department of Biostatistics, 1161 21st Avenue South D-2220 Medical Center North, Nashville, TN, 37232-2158, USA; 4 Vanderbilt University Medical Center, Division of Critical Care, Department of Anesthesia, 1211 21st Avenue South, 526 Medical Arts Building, Nashville, TN, 37212, USA

**Keywords:** Traumatic brain injury, Sympathetic hyperactivity, Sympathetic storm, Autonomic dysfunction, Adrenergic blockade, Beta-blocker, Alpha_2_-agonist, Propranolol, Clonidine, Agitation

## Abstract

**Background:**

Severe TBI, defined as a Glasgow Coma Scale ≤ 8, increases intracranial pressure and activates the sympathetic nervous system. Sympathetic hyperactivity after TBI manifests as catecholamine excess, hypertension, abnormal heart rate variability, and agitation, and is associated with poor neuropsychological outcome. Propranolol and clonidine are centrally acting drugs that may decrease sympathetic outflow, brain edema, and agitation. However, there is no prospective randomized evidence available demonstrating the feasibility, outcome benefits, and safety for adrenergic blockade after TBI.

**Methods/Design:**

The DASH after TBI study is an actively accruing, single-center, randomized, double-blinded, placebo-controlled, two-arm trial, where one group receives centrally acting sympatholytic drugs, propranolol (1 mg intravenously every 6 h for 7 days) and clonidine (0.1 mg per tube every 12 h for 7 days), and the other group, double placebo, within 48 h of severe TBI. The study uses a weighted adaptive minimization randomization with categories of age and Marshall head CT classification. Feasibility will be assessed by ability to provide a neuroradiology read for randomization, by treatment contamination, and by treatment compliance. The primary endpoint is reduction in plasma norepinephrine level as measured on day 8. Secondary endpoints include comprehensive plasma and urine catecholamine levels, heart rate variability, arrhythmia occurrence, infections, agitation measures using the Richmond Agitation-Sedation Scale and Agitated Behavior scale, medication use (anti-hypertensive, sedative, analgesic, and antipsychotic), coma-free days, ventilator-free days, length of stay, and mortality. Neuropsychological outcomes will be measured at hospital discharge and at 3 and 12 months. The domains tested will include global executive function, memory, processing speed, visual-spatial, and behavior. Other assessments include the Extended Glasgow Outcome Scale and Quality of Life after Brain Injury scale. Safety parameters evaluated will include cardiac complications.

**Discussion:**

The DASH After TBI Study is the first randomized, double-blinded, placebo-controlled trial powered to determine feasibility and investigate safety and outcomes associated with adrenergic blockade in patients with severe TBI. If the study results in positive trends, this could provide pilot evidence for a larger multicenter randomized clinical trial. If there is no effect of therapy, this trial would still provide a robust prospective description of sympathetic hyperactivity after TBI.

**Trial registration:**

ClinicalTrials.gov NCT01322048

## Background

In developed countries, traumatic brain injury (TBI) is the leading cause of death and disability among young adults. In the United States alone, TBI affects more than 2 million individuals annually, and the direct and indirect annual costs related to TBI are estimated at $56 billion. Each year, TBI results in 50,000 deaths and 80,000 survivors suffer from long-term disability [1,2]. Severe TBI, defined as a Glasgow Coma Scale (GCS) ≤ 8, is associated with increased intracranial pressure and activates the sympathetic nervous system, resulting in an increase in plasma catecholamine levels.

There is a direct correlation between severe TBI and this catecholamine surge [3]. Immediately after TBI, plasma epinephrine and norepinephrine levels increase several-fold, and remain elevated in those who have persistent coma or are moribund [4-6]. Those with initial catecholamine levels that are only mildly elevated have been found to improve to a GCS > 11 at 1 week. In those with multisystem trauma and TBI, plasma norepinephrine levels at 48 h post injury are predictive of GCS at 1 week, survival, number of ventilator days, and the length of stay (LOS); without TBI, these associations were absent [7].

**Figure 1:**
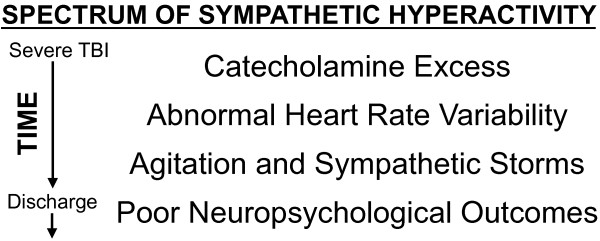
**Conceptual model of sympathetichyperactivity after severe TBI**.

Systemic manifestations of this sympathetic surge include paroxysms of tachycardia, tachypnea, hypertension, and hyperpyrexia with associated motor features such as agitation and dystonia [8]. Our group has shown that increased TBI severity also correlates with decreased heart rate variability (HRV); this is another reflection of autonomic dysfunction [9-11]. Clinically, these ill-defined intermittent episodes, often a diagnosis of exclusion, are termed ‘sympathetic storms’ or ‘autonomic storms’, frequently manifest with “aggression”, or “agitation”. [12,13]. This is most prevalent during the acute stage of recovery, particularly at coma emergence, with reported incidence rates up to 96% [14]. Notably, persistent sympathetic hyperactivity is associated with increased intensive care unit (ICU) LOS, lower cognitive ability, and higher cognitive fatigue [15-17]. While the full spectrum of sympathetic hyperactivity after TBI has not been systematically described nor intervened upon, our hypothesized model based on literature review and clinical experience is shown in Figure 1.

One strategy to decrease sympathetic hyperactivity is pharmacologic intervention with beta (β)-blockade. Non-selective β-blockade with propranolol, in pre-clinical mouse models, reduces brain edema, improves neurologic outcomes [18], increases cerebral perfusion [19], and decreases cerebral hypoxia [20]. Also, propranolol can reduce the maximum intensity of agitated episodes [14], and even reduces aggressive behavior months after TBI [21,22]. This work has led to two parallel, non-placebo-controlled, open-label, prospective, single-center studies (NCT01202110, NCT01343329), which employ early propranolol after TBI and monitor short-term endpoints, like heart rate [23].

Several retrospective studies [24,25], including two from our group [9,26], have indicated that β-blockade exposure following TBI conveys a 4% to 23% absolute mortality advantage [3]. This mortality benefit is even larger if stratified by early physiological measures of sympathetic excess, such as decreased HRV. Though these findings have resulted in an increase in β-blocker use in our institution from 20% to over 40% in young, severe TBI patients over a 5-year period [9], rigorous prospective evidence regarding the feasibility, outcome benefits and safety of using of β-blockers in TBI patients is lacking.

β-blockade is just one pharmacologic strategy to reduce sympathetic hyperactivity; centrally acting alpha_2_ (α_2_)-agonists also serve as sympatholytic agents [27-29]. The prototypical centrally acting α_2_-agonist, clonidine, decreases plasma catecholamines and improves outcomes in a rat model of incomplete cerebral ischemia [30]. Clinically, clonidine decreases plasma catecholamines and cerebral vasoconstriction without altering cerebral blood flow in patients with severe TBI [31,32].

Strong clinical data from Sweden suggest that limiting the adrenergic response after severe TBI in patients with concurrent use of metoprolol and clonidine limits the formation of cerebral edema. Although the reduction in mean arterial blood pressure may lower cerebral perfusion pressure, this group has hemodynamic and brain microdialysis data showing that the use of metoprolol and clonidine is well tolerated by TBI patients with a neurologic and mortality benefit [33,34]. The Lund neurotrauma physicians in Sweden are pioneers in the nonsurgical reduction of increased intracranial pressure after severe TBI, and combined adrenergic blockade is standard of care in their TBI protocols. Even when studied outside of Sweden, the Lund concept showed better outcomes when tested against standard of care among a mixed population of aneurysmal subarachnoid hemorrhage and TBI [35].

Clonidine and propranolol are lipophilic, penetrate the blood brain barrier, and are used to address the paroxysmal agitation associated with TBI [11,12]. Both drugs have variable effects on memory, emotion, and cognition [36-39]; however, these effects are not defined after TBI. Although the above European data have shown stable cerebral perfusion pressure when using these agents, the early empiric use of these anti-hypertensive agents is considered innovative within North American TBI environments, where feasibility and safety are not clear. Furthermore, because both clonidine and propranolol may ameliorate the spectrum of sympathetic hyperactivity after TBI, and using both drugs within common dosage frequencies would provide multiple treatment delivery opportunities per day within a complex ICU environment, we choose to study both drugs as a treatment combination.

Using our actively accruing, single-center, double-blinded, placebo-controlled, randomized clinical trial (RCT), the DASH After TBI Study, we intend to determine the effect of combined adrenergic blockade using propranolol and clonidine on: (1) short-term physiology, behavior, and cognition; and (2) long-term neuropsychological outcomes after severe TBI.

## Methods and design

### Objectives and design

**Figure 2:**
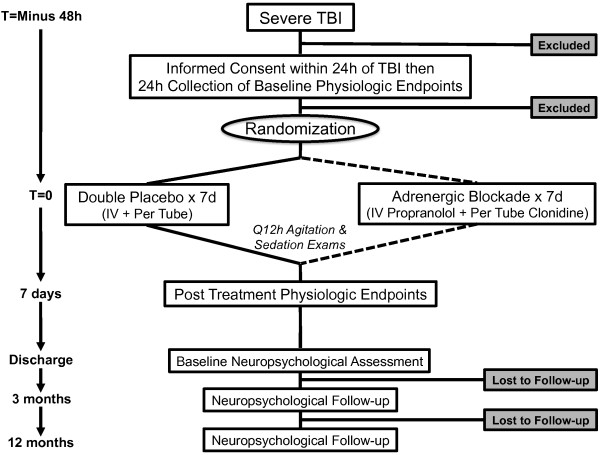
**CONSORT diagram of theDASH After TBI Study**.

The DASH after TBI study is an actively accruing, single-center, randomized, double-blinded, placebo-controlled trial, where one group receives centrally acting sympatholytic drugs, propranolol and clonidine, and the other group, double placebo, within 48 h of severe TBI. Our primary hypothesis is that adrenergic blockade after severe TBI will be associated with decreased catecholamine levels, normalization of HRV, and decreased autonomic response to cold pressor testing. Figure 2 shows the CONSORT [40] diagram of the DASH After TBI Study.

### Study population

The trial setting is Vanderbilt University Medical Center and patients are screened and enrolled in an ICU environment. Inclusion criteria are severe TBI (GCS ≤ 8) with injury on CT, ages 16 to 64 years, and screen completed within 24 h of injury. Exclusion criteria are listed in Table 1. Both inclusion and exclusion criteria are similar to clinical trials in severe TBI. Proxy consent is obtained from next of kin. If kin cannot be found, the patient is ineligible and no emergency consent is used.

**Table 1:** Exclusion criteria

	
**Pre-existing condition**	
	Heart disease
	Cardiac dysrhythmia
	Allergy to study drugs
**Brain-related**	
	Penetrating brain injury
	Pre-existing brain dysfunction
	Impending brain herniation
	Craniectomy or craniotomy
**Physiologic**	
	Spinal cord injury
	Myocardial injury
	Severe liver disease
	Current use of β-blocker and/or α_2_-agonist
	Withdrawal of care expected in 24 h
**Demographic**	
	Prisoners
	Pregnant women
	Unable to follow-up through final visit

### Randomization

To maintain group balance among several factors, treatment group allocation is determined using weighted minimization randomization with a random element [41]. The factors we are balancing are age and severity of brain injury, using Marshall Head computed tomography (CT) classifications [42] read by a neuroradiologist into one of the following categories: CT class II (cisterns open); CT class III (swelling); CT class IV (midline shift); CT Class VI (non-evacuated mass lesion). Of note, we are tracking the feasibility of being able to provide a neuroradiology read for randomization within 24 h of consent. The age factor is weighted twice as much as Marshall classification, and is defined separately for each new patient as the patient’s age ± 5 years. The randomization program incorporates a random element in the following way: with probability of 0.75, or 75% of the time, each new patient is assigned to the treatment that best balances the two groups given the current makeup of the two groups. Using this probability of 0.75 rather than always assigning to achieve balance allows there to be a random and, thus, less predictable element, while still achieving balance between the two groups for these factors. The randomization program was created in R version 2.14.0 [43].

For each new patient, personnel at the investigational pharmacy enter the new patient’s age and Marshall class using a dedicated, password-protected, randomization website. The website interfaces with rApache software [44] and the algorithm written in R determines the treatment allocation. The treatment assignment is then saved to a database and returned to the screen, indicating which treatment for the pharmacy to provide for the current patient. Study biostatisticians monitor the group assignments for different types of imbalances and can intervene and assign deterministically (in a non-random way) to balance the treatment groups in the case of extreme imbalance.

Without accounting separately in the randomization algorithm for imbalances in group size, simulation of the adaptive randomization program shows 93% of the time, the differences between groups will be ≤4, and the average group difference will be zero, thus approximating a 1:1 randomization ratio.

### Treatment delivery

Study drugs start within 48 h of injury but after plasma and 24-h urine catecholamine baseline measurements. Both drugs (or double placebos) are administered for 7 days. Propranolol is administered intravenously at a dose of 1 mg every 6 h, and doses are held for heart rate less than 60 bpm, mean arterial pressure less than 60 mmHg, or cerebral perfusion pressure less than 60 mmHg. Clonidine is administered at a dose of 0.1 mg per tube every 12 h, and is held for mean arterial pressure <60 mmHg, or cerebral perfusion pressure <60 mmHg. Administration of propranolol and clonidine, or of the double placebos, is staggered. If hemodynamic parameters are met while a patient is on pressors, drug delivery still occurs. Other aspects of study feasibility being documented are compliance with treatment delivery and reasons for withholding treatment.

### Protocol contamination and/or dropout

Clinicians are allowed to use β-blockers at any point if there is myocardial infarction or need for heart rate control that is refractory to calcium channel blockers and/or anti-arrhythmic medications. Dexmedetomidine, a prototypical α_2_-agonist, is allowed after failure of standard sedative regimens like propofol, lorazepam, or midazolam. Any β-blocker or α_2_-agonist is allowed after the post-treatment plasma and 24-h urine catecholamine assessments. Complete follow-up is always performed and the planned analysis is intention to treat.

Other protocol directives to decrease confounding in the catecholamine endpoints are the preferred pressors of phenylephrine, vasopressin, dobutamine, and milrinone. Norepinephrine, epinephrine, and dopamine agents are to be avoided unless increased cardiac output is needed or further systemic vascular resistance is required beyond that produced by preferred pressors. The feasibility of avoiding non-study sympatholytic drug use, which results in treatment contamination, is also being tracked.

### Study endpoints

We collect baseline data regarding demographics, socioeconomic status, medical history, medication use, and injury characteristics and severity, according to the common data elements for TBI advocated by multiple agencies, including the National Institute of Neurological Disorders and Stroke. Also, we collect pathoanatomic data elements that encompass the Marshall CT classification, Rotterdam CT score, and other consensus-derived data elements [45,46].

Our current primary endpoint is plasma norepinephrine level on day 8. Within 1 h of enrollment and after treatment on day 8, blood is drawn for plasma catecholamine measures and a 24-h urine collection is started for urine catecholamine measures. Blood is collected into cooled heparinized tubes, which are immediately placed on ice until they are centrifuged at for 10 min at 3,000 rpm. Plasma is harvested and stored in tubes containing 40 mL of reduced glutathione (6%) at −20°C until it is assayed. Catecholamine concentrations are measured by high performance liquid chromatography using electrochemical detection with dihydroxybenzylamine as the internal standard [47]. Catecholamines measured include norepinephrine, epinephrine, dopamine, dihydroxyphenylglycol, dihydroxyphenylalanine, and dihydroxyphenylacetic acid.

Other physiologic measures of response for the DASH After TBI Study are HRV and related responses to autonomic cold pressor testing, daily physiologic measures (for example, temperature, blood pressure, heart rate, intracranial pressure), arrhythmia occurrence, infections, adverse and serious adverse events.

Additional secondary outcomes are twice daily measurements of the Richmond Agitation-Sedation Scale (RASS) and Agitated Behavior Scale (ABS) for TBI, daily medication use (anti-hypertensives, sedatives, analgesics, and antipsychotics), coma-free days, ventilator-free days, ICU LOS, hospital LOS, and in-hospital mortality.

A long-term study component begins with a baseline neuropsychological evaluation performed at hospital discharge. In the Vanderbilt Multidisciplinary TBI Clinic at 3 and 12 months, neuropsychological tests cover global executive function, memory, processing speed, visual-spatial, and behavioral domains, in addition to the validated Extended Glasgow Outcome Scale (GOSE) and (Quality of Life after Brain Injury) QOLIBRI scales. The specific neuropsychological tests are as follows: Tower Test, Galveston Orientation and Amnesia Test, Numbers Reversed and Verbal Analogies from Woodcock-Johnson: Tests of Cognitive Abilities, Three Word Recall from Modified Orientation Amnesia Test, Story Retelling Immediate Subtest and Delayed Subtest from Arizona Battery for Communication Disorders of Dementia, Trail Making A&B, Stroop Test, Word Fluency, and Zoo Map from Behavioral Assessment of Dysexecutive Syndrome. Also, the Social Security Death Index is queried monthly for long-term mortality assessments.

Safety parameters collected throughout the study include cardiac complications such as dysrhythmia (for example, symptomatic bradycardia), myocardial infarction, and cardiac arrest. Serious adverse events are reported in a blinded fashion to the Data Safety Monitoring Board within 24 h.

### Data management

Vanderbilt University, with collaboration from a consortium of institutional partners, has developed a software toolset and workflow methodology for electronic collection and management of research and clinical trial data called REDCap (Research Electronic Data Capture) [48]. REDCap servers are housed in a local data center at Vanderbilt, and all web-based information transmission is encrypted. All current protocols, consent forms, and data are stored in REDCap.

### Current sample size justification

Primarily, we expect plasma norepinephrine reduction after adrenergic blockade in severe TBI subjects. This continuous response variable will involve an independent control and experimental subjects with one control per experimental subject. In previous TBI studies [3,6], the baseline plasma norepinephrine level was 1,686 ± 416 pg/mL. If the plasma norepinephrine level decreases to 1,236 pg/mL after adrenergic blockade, then the true difference in the experimental and control means is 450 pg/mL. Using the Power and Sample Size Calculation program [49], we will need to study 19 experimental subjects and 19 control subjects to be able to reject the null hypothesis that the population means of the experimental and control groups are equal with a probability (power) of 0.9. The Type I error probability with testing this null hypothesis is 0.05.

**Figure 3:**
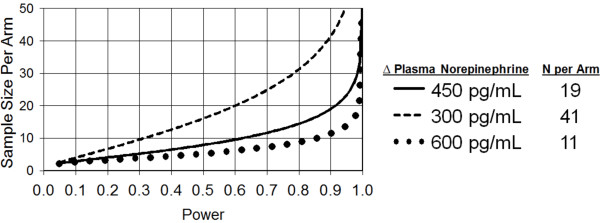
**Current sample size *vs. *power for Δ plasma norepinephrine on day 8**.

If the absolute reduction of plasma norepinephrine using adrenergic blockade after severe TBI is only 300 pg/mL, and power is constant at 0.9 and the Type I error probability remains at 0.05, then we will need to study 41 patients per arm. Alternatively, if plasma norepinephrine is reduced by 600 pg/mL after intervention, then we will only need to study 11 patients per arm (Figure 3).

Our center admits over 200 severe TBI patients per year, but given the strict eligibility criteria, and assuming a conservative 10% enrollment rate, we anticipate a 24-month total accrual time for 38 patients. An additional 12-month follow-up is required to fulfill all of the DASH After TBI Study aims. Lastly, despite exclusion criteria, we assume a 5% early mortality (<1 week) related to critical illness. Accordingly, our current sample size is 20 patients per arm or a total of 40 patients.

### Statistical analysis

All analyses will occur on an intention to treat basis and will be blinded to treatment assignment. Interim analysis is not planned, unless the sample size is expanded. The primary endpoint is post-treatment plasma norepinephrine level on day 8. The outcome is continuous and independent, while the exposure is dichotomous. To test for an association between post-treatment plasma norepinephrine level and treatment, we will use a Wilcoxon rank sum test statistic with the standard error determined empirically by the randomization method.

HRV linear analyses (frequency and spectral) will be performed. HRV spectral analysis will be performed to calculate very low frequency, low frequency (LF), and high frequency (HF) bands, with a LF/HF ratio calculation to understand sympathovagal balance. Understanding the limitations of spectral analysis, we will compute other HRV measurements including standard deviation of normal beat intervals and entropy.

For secondary outcome analyses, the Type I error probability will be adjusted to account for multiple comparisons. We will use Fisher’s exact test to compare categorical variables between the study groups (for example, RASS). GOSE will be compared using an ordinal approach of sliding dichotomy [50]; QOLIBRI, ventilator-free days, hospital LOS, and ICU LOS are continuous measures that will be analyzed the same as the primary endpoint. Mortality will be studied using Kaplan-Meier analysis and Cox Proportional Hazard Modeling. The raw test scores of the neuropsychological battery will be transformed into standardized scores based on mean and standard deviations of the normative sample with similar age and education levels (T-scores). These T-scores will be summarized using medians with interquartile ranges for continuous outcome variables, and frequencies or proportions for categorical outcome variables.

### Ethics

The DASH After TBI Study is conducted in accordance with the Declaration of Helsinki and was approved by the Vanderbilt University Medical Center Institutional Review Board. Informed consent is obtained from patient surrogates and subsequently from patients, if they have neurologically recovered. The DASH After TBI Study is registered with ClinicalTrials.gov by identification number NCT01322048.

## Discussion

The DASH After TBI Study is the first randomized, double-blinded, placebo-controlled trial powered to investigate combined adrenergic blockade in patients with severe TBI. If the DASH After TBI Study produces positive trends, this could provide pilot evidence for an entire class of neuroprotective agents and open doors for a larger multicenter RCT. If there is no effect of therapy, this trial would still provide a robust prospective description of sympathetic hyperactivity after TBI.

This feasibility RCT has some unique features that may be helpful for future TBI trial design. This study uses an adaptive co-variate randomization to achieve balance between important co-variates of age and Marshall CT class in a small sample size setting. Although we have one neuroradiologist providing head CT characteristics for patient enrollment stratification, there are two other neuroradiologists providing blinded head CT readings, thus creating a nested prospective reliability study of using Marshall CT class for randomization inputs into a time-sensitive TBI trial. Another unique feature is the broad 24-h window for enrollment that is much longer than most acute TBI trials. This time window allows common toxic-metabolic reasons for coma to be ruled out, as well as excluding those patients who are moribund from any cause. Also, this RCT may define the drug delivery compliance within the context of our protocol and patient physiology. Although our doses are common starting ranges and frequencies for these medications and will diminish the chance hemodynamic responses will break the blind, we are assessing treatment delivery in detail.

This RCT uses combined adrenergic blockade, which obscures whether any effect is mediated by α or β-receptor mechanisms. We chose both drugs due to the clinical ubiquity of combined use and to provide a higher chance of detectable endpoints for a small trial. Also, the optimal duration of therapy is unclear. However, we felt it is safest and most feasible to restrict therapy to 7 days, when patients would be monitored closely in the ICU. Using an intravenous, centrally acting α_2_-agonist, such as dexmedetomidine, would provide more reliable drug delivery and potential therapeutic response, but this is a risky approach for a pilot RCT.

Due to the physical brain damage after severe TBI, we excluded delirium from our cognitive assessments. Contemporary delirium metrics would be positive for 100% of severe TBI patients for weeks, if not months. Sedatives are a risk factor for delirium and cognitive dysfunction in the ICU, and they may play a stronger role on outcomes than the adrenergic therapy. Although sedation guidelines after severe TBI are not defined, our study does have a standardized sedation protocol, and we are measuring sedative use.

Having intense neurologic monitoring for intracranial pressure, local cerebral oxygenation and metabolism, cerebral blood flow, and/or white matter tract imaging would be ideal, but none are standard of care. In future studies, we may use MRI with diffuse tensor imaging protocols to non-invasively assess white tract matter damage and recovery, as there is a link between deeper intraparenchymal brain lesions and sympathetic hyperactivity [51].

At the present time, we do not have a planned interim analysis, though all serious adverse events are reported to our data safety monitoring board. Given our current sample size of 40, an interim analysis would not show early dramatic benefits or provide a reason for early trial termination. If we obtain further funding and expand our current sample size from 40 patients, then we will perform a group sequential interim analysis using the O’Brien-Fleming method. For example using the same primary endpoint, if sample size is expanded to 80 patients, we are prepared for an *ad-hoc* subgroup analysis where age is dichotomized using the median, such that 38 experimental subjects and 38 control subjects will be required. This also allows for an *ad-hoc* analysis of outcome by combined Marshall head CT classes II & III. Furthermore, this expanded sample size would also permit over an 80% power to detect a 2-day difference in the secondary endpoint of ventilator-free days. Notably, ventilator-free days also represents a potentially viable primary endpoint to shift towards, given its better external generalizability, at the possible expense of an increased sample size.

In conclusion, the DASH After TBI Study is an investigator-initiated, single-center, double-blinded, placebo-controlled RCT powered to test the hypothesis that combined adrenergic blockade after severe TBI will decrease plasma norepinephrine levels. This study also determines the effect of adrenergic blockade on ICU metrics, like sedation efficacy and agitation measures, as well as long-term neuropsychological outcomes after severe TBI.

## Trial status

The DASH After TBI study was registered on March 2, 2011 at http://clinicaltrials.gov and its trial identifier is NCT01322048. The Vanderbilt University Institutional Review Board approved the study protocol on May 27, 2011. The study is conducted in accordance with Good Clinical Practice Guidelines. Screening began on August 9, 2011, and the first patient was randomized on August 23, 2011. For the current sample size, the recruitment period is planned until August 2013 with follow-up concluding in August 2014.

## Abbreviations

ABS: Agitated behavior scale; α_2_: Alpha_2_; β: Beta; CT: Computed tomography; DASH After TBI: Decreasing adrenergic or sympathetic hyperactivity after severe traumatic brain injury; GCS: Glasgow coma scale; GOSE: Extended glasgow outcome scale; HF: High frequency; HRV: Heart rate variability; ICU: Intensive care unit; LF: Low frequency; LOS: Length of stay; QOLIBRI: Quality of life after brain injury; RASS: Richmond agitation-sedation scale; RCT: Randomized clinical trial; REDCap: Research electronic data capture; TBI: Traumatic brain injury.

## Competing interests

The authors declare that they have no competing interests.

## Authors’ contributions

MBP designed the study, wrote the protocol drafts, developed the randomization scheme, planned the statistical analysis, received peer-reviewed funding for the study, coordinates the trial, enrolls patients, acquires trial data, drafted the manuscript, and is implementing the study. JMÁ designed, programmed, and implemented the weighted adaptive minimization randomization program, coordinated the web-based conversion, monitors for group imbalance during accrual, and drafted the statistical methods of the manuscript. ODG helped design the study, acquires trial data, revised protocol drafts, and reviewed initial drafts of the manuscript. JWM and AS enroll patients, acquire trial data, revised protocol drafts, and reviewed initial drafts of the manuscript. JMJ and PPP helped design the study, acquire trial data, revised protocol drafts, reviewed initial drafts of the manuscript, and provide oversight on trial coordination and implementation. All authors approved the final version of the manuscript.

## Authors’ information

MBP is an Assistant Professor of Surgery and Neurosurgery, trauma surgeon and surgical critical care intensivist at the Nashville VAMC (Surgery service) and at VUMC. ODG is an Associate Professor of Surgery and Neurosurgery, Director of the VUMC Trauma ICU, trauma surgeon, and surgical critical care intensivist. JMA is a VUMC Biostatistician III and member of the Bayesian Adaptive Trial Design Workforce. JMJ is our senior clinical research nurse for the VUMC Division of Trauma & Surgical Critical Care. JWM and AS are pre-doctoral trauma research trainees. PPP is a critical care intensivist at the Nashville VAMC (Anesthesia service), VUMC Associate Professor of Anesthesiology, Critical Care, and Surgery, and is site co-PI for the ICU Delirium and Cognitive Impairment Study Group.
